# 1,25-Dihydroxyvitamin D_3_ Inhibits the Differentiation and Migration of T_H_17 Cells to Protect against Experimental Autoimmune Encephalomyelitis

**DOI:** 10.1371/journal.pone.0012925

**Published:** 2010-09-23

**Authors:** Jae-Hoon Chang, Hye-Ran Cha, Dong-Sup Lee, Kyoung Yul Seo, Mi-Na Kweon

**Affiliations:** 1 Mucosal Immunology Section, Laboratory Science Division, International Vaccine Institute, Seoul, Korea; 2 Cancer Research Institute, Seoul National University College of Medicine, Seoul, Korea; 3 Department of Ophthalmology, Institute for Vision Research, Yonsei University College of Medicine, Seoul, Korea; New York University, United States of America

## Abstract

**Background:**

Vitamin D_3_, the most physiologically relevant form of vitamin D, is an essential organic compound that has been shown to have a crucial effect on the immune responses. Vitamin D_3_ ameliorates the onset of the experimental autoimmune encephalomyelitis (EAE); however, the direct effect of vitamin D_3_ on T cells is largely unknown.

**Methodology/Principal Findings:**

In an *in vitro* system using cells from mice, the active form of vitamin D_3_ (1,25-dihydroxyvitamin D_3_) suppresses both interleukin (IL)-17-producing T cells (T_H_17) and regulatory T cells (Treg) differentiation via a vitamin D receptor signal. The ability of 1,25-dihydroxyvitamin D_3_ (1,25(OH)_2_D_3_) to reduce the amount of IL-2 regulates the generation of Treg cells, but not T_H_17 cells. Under T_H_17-polarizing conditions, 1,25(OH)_2_D_3_ helps to increase the numbers of IL-10-producing T cells, but 1,25(OH)_2_D_3_'s negative regulation of T_H_17 development is still defined in the IL-10^−/−^ T cells. Although the STAT1 signal reciprocally affects the secretion of IL-10 and IL-17, 1,25(OH)_2_D_3_ inhibits IL-17 production in STAT1^−/−^ T cells. Most interestingly, 1,25(OH)_2_D_3_ negatively regulates CCR6 expression which might be essential for T_H_17 cells to enter the central nervous system and initiate EAE.

**Conclusions/Significance:**

Our present results in an experimental murine model suggest that 1,25(OH)_2_D_3_ can directly regulate T cell differentiation and could be applied in preventive and therapeutic strategies for T_H_17-mediated autoimmune diseases.

## Introduction

Interleukin (IL)-17-producing T cells have been identified in the mouse as a new lineage of CD4^+^ T cells that can be differentiated from naïve T cells by the polarizing cytokines TGF-β, IL-6, and IL-23 [1]–[4]. T_H_17 cells can protect against bacterial pathogens by recruiting neutrophils but have also been reported to develop into an immunopathology in various models of autoimmunity [1]–[4].

Multiple sclerosis (MS) is a chronic autoimmune disease of the central nervous system (CNS) characterized by inflammatory cell infiltration and subsequent demyelination of axonal tracts in the brain and spinal cord [5]. Demyelination disturbs the conduction of neuronal signals along axons, resulting in clinical symptoms including pain, fatigue, muscle weakness, and visual disturbances [5]. Several studies report that T_H_17 cells are involved in the initiation and maintenance of experimental autoimmune encephalomyelitis (EAE), a murine model of MS [6], [7]. In addition, recent studies suggest that T_H_17 cells (i.e., IL-17^+^ T_H_17 cells) have a high inflammatory potential and may constitute a relevant inflammatory subset in human MS [8], [9]. Some of these T_H_17 cells secrete IFN-γ (i.e., IFN-γ^+^ T_H_17 cells), which preferentially migrates into the CNS in human MS [10], [11].

Although the exact cause of MS remains unclear, genetic background and/or unknown environmental factors are believed to contribute to the onset of the disease. Epidemiological studies have shown that geographical location is associated with the incidence of MS, which increases with latitude in both hemispheres [12]. One potential explanation is that susceptibility to MS is related to exposure to sunlight and the subsequent production of vitamin D [13]. In one recent study, levels of vitamin D were significantly lower in relapsing-remitting patients than in healthy controls [14]. In addition, the level of vitamin D production in MS patients suffering a relapse was lower than in patients during remission [14]. Furthermore, vitamin D supplementation and higher levels of vitamin D in circulation are associated with a decreased incidence of MS [15], [16].

Vitamin D is a well-known nutrient that acts as a modulator of calcium homeostasis and the immune response [17], and the vitamin D receptor (VDR) is expressed in several types of immune cells, including monocytes, macrophages, dendritic cells (DCs), and effector/memory T cells [18]–[20]. In *in vitro* studies, 1,25(OH)_2_D_3_ inhibits T cell proliferation, the production of IL-2 and IFN-γ and cytotoxicity [21]–[23]. 1,25(OH)_2_D_3_ negatively regulates the differentiation, maturation, and immunostimulatory capacity of DCs by decreasing the expression of MHC class II, CD40, CD80, and CD86 [24]–[26]. In addition, 1,25(OH)_2_D_3_ decreases the synthesis of IL-6, IL-12, and IL-23 [27]–[29]. Hence it seems likely that 1,25(OH)_2_D_3_ suppresses the generation of T_H_1 and T_H_17 cells and probably induces the development of forkhead box protein 3 (Foxp3)^+^ Treg cells. However, the direct effect of 1,25(OH)_2_D_3_ on the function and differentiation of T cells is largely unknown because VDR is not expressed in naïve T cells [30]. Thus, these inhibitory effects of 1,25(OH)_2_D_3_ are most pronounced in the effector/memory T cells which do express VDR or are mediated by 1,25(OH)_2_D_3_-treated DCs.

In this study, we addressed whether 1,25(OH)_2_D_3_ directly down-regulates the development of both Treg and T_H_17 cells. These inhibitory capabilities of 1,25(OH)_2_D_3_ are dependent on the VDR signal in activated CD4^+^ T cells. Importantly, 1,25(OH)_2_D_3_ regulates the migration of T_H_17 cells into the CNS by suppressing CCR6 expression. Our findings establish that oral treatment with systemic 1,25(OH)_2_D_3_ directly modulates to T cells to prevent both the development of T_H_17 cells and the expression of CCR6 in EAE-induced conditions. Therefore, vitamin D_3_ could be applicable in both preventive and therapeutic strategies for T_H_17-mediated autoimmune disease.

## Results

### 1,25(OH)_2_D_3_ inhibits the onset of EAE and alters T_H_ cell composition

**Figure 1:**
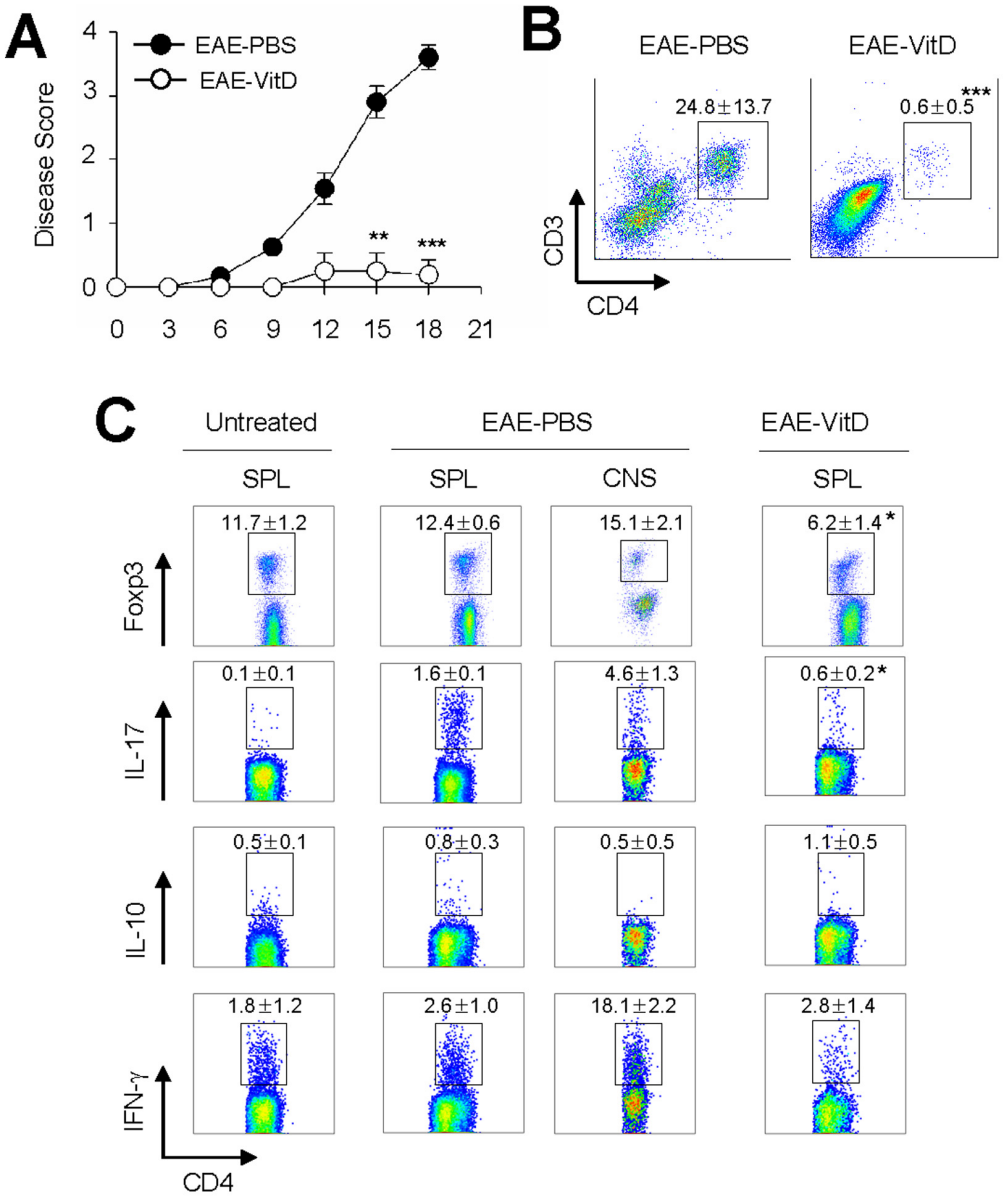
1,25(OH)_2_D_3_ inhibits the onset of EAE and modulates the composition of T_H_ cells. (**A**) Disease scores are shown for EAE in B6 mice at various time points after subcutaneous immunization with MOG_35–55_ peptide in CFA and pertussis toxin. Results shown are mean ± SD. **p<0.01, ***p<0.001, compared with EAE-PBS group. (**B**) At 20 days after challenge, total mononuclear cells obtained from the brains of MOG_35–55_-immunized wild-type mice and vitamin D_3-_treated mice and stained with anti-CD4 and anti-CD3 Abs. Data are representative of three independent experiments with at least five mice per group. ***p<0.001, compared with EAE-PBS group. (**C**) Mononuclear cells from brains or splenocytes were restimulated *in vitro* with PMA/ionomycin for 5 hr, then stained intracellularly for Foxp3, IL-17A, IL-10, and IFN-γ. Data are representative of three independent experiments with at least five mice per group. *p<0.05, compared with splenocytes of EAE-PBS group.

To develop an animal experimental model of EAE, B6 mice were immunized subcutaneously with a peptide consisting of myelin oligodendrocyte glycoprotein (MOG_33–55_) in complete Freund's adjuvant (CFA) and pertussis toxin as described elsewhere [31]–[34]. The severity of the resulting paralysis was determined as a disease score. Symptoms were shown at 9 days after challenge and high severity of paralysis was shown at about 20 days (Figure 1A). To confirm whether vitamin D_3_ inhibits EAE initiation, mice were orally treated with 1,25(OH)_2_D_3_ as described elsewhere [32]. Of note, most 1,25(OH)_2_D_3_-treated mice were completely resistant to the development of EAE (Figure 1A). Since previous studies demonstrated that autoreactive T cells, especially T_H_1 and T_H_17, are essential to induce EAE, we further analyzed T_H_ cells in EAE-induced mice. To this end, mononuclear cells in the CNS (including the brain and spinal cord) were enriched by density gradient and analyzed by flow cytometry. As depicted in Figure 1B, significantly fewer infiltrated CD4^+^ T cells were present in the CNS of the 1,25(OH)_2_D_3_-treated EAE-induced mice than in the CNS of PBS-treated EAE-induced mice. We further analyzed the T_H_ differentiation in the spleen and CNS of EAE-induced mice with and without oral 1,25(OH)_2_D_3_. As expected, IL-17-secreting T_H_17 cells were predominant in the spleen of EAE-induced mice when compared with the untreated wild-type B6 mice (Figure 1C). Of note, oral treatment with 1,25(OH)_2_D_3_ dramatically reduced the numbers of T_H_17 cells in the spleen of EAE-induced mice (Figure 1C, *p* = 0.00114). In addition, increased numbers of T_H_17 cells were detected in the CNS of EAE-induced mice (Figure 1C) whereas no T_H_17 cells were detected in the CNS of 1,25(OH)_2_D_3_-treated mice (data not shown). The number of Foxp3^+^ cells in the spleen of 1,25(OH)_2_D_3_-treated mice was slightly decreased, but the numbers of IL-10 and IFN-γ expressing cells in the spleen of all groups of mice were identical. Taken together, these results suggest that vitamin D_3_ may regulate the differentiation and/or migration of CD4^+^ T cells in the EAE inductive phase.

### 1,25(OH)_2_D_3_ inhibits in vitro differentiation of both Treg and T_H_17 cells

**Figure 2:**
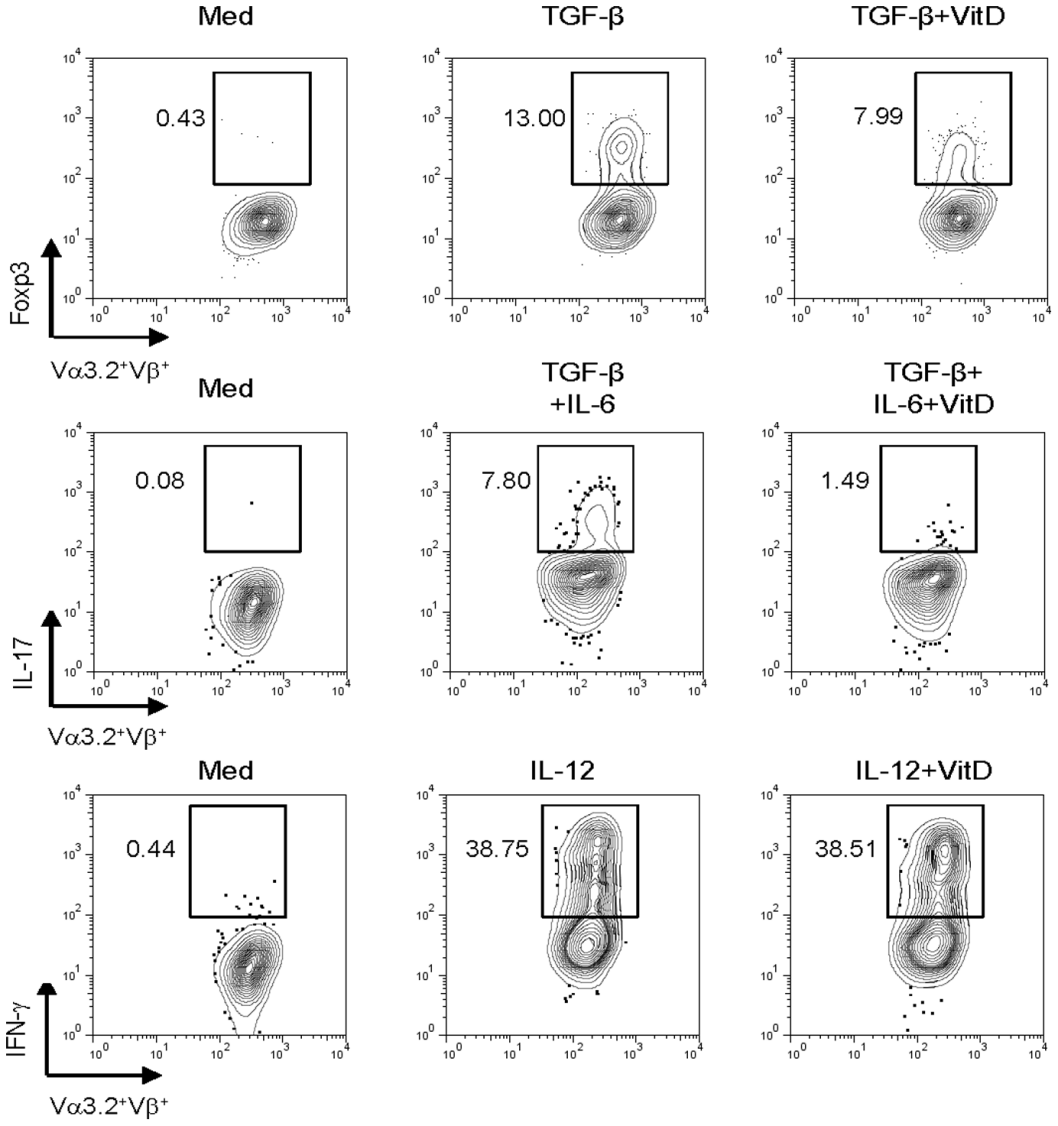
1,25(OH)_2_D_3_ negatively regulates Treg and T_H_17 induction in neuro-antigen-specific CD4^+^ T cells. CD4^+^ T cells isolated from MOG TCR-Tg mice (Vα3.2 and Vβ11 TCR, B6 background) were cultured with MOG_35–55_ peptide (25 µg/ml) in the presence of CD3^+^ T cell-depleted splenocytes for 4 days under Treg-polarizing conditions (rTGF-β, 1 ng/ml; anti-IFN-γ, 10 µg/ml; and anti-IL-4, 10 µg/ml) or T_H_17-polarizing conditions (rTGF-β, 1 ng/ml; rIL-6, 20 ng/ml; anti-IFN-γ, 10 µg/ml; and anti-IL-4, 10 µg/ml) or T_H_1-polarizing conditions (rIL-12, 10 ng/ml; and anti-IL-4, 10 µg/ml) together with 1,25(OH)_2_D_3_ (VitD, 100 nM). Cells were then stained intracellularly for Foxp3, IL-17, or IFN-γ, respectively. The plots shown are gated on CD4^+^Vα3.2^+^ cells with quadrants drawn based on isotype controls. Data are representative of two independent experiments with at least three mice per group.

**Figure 3:**
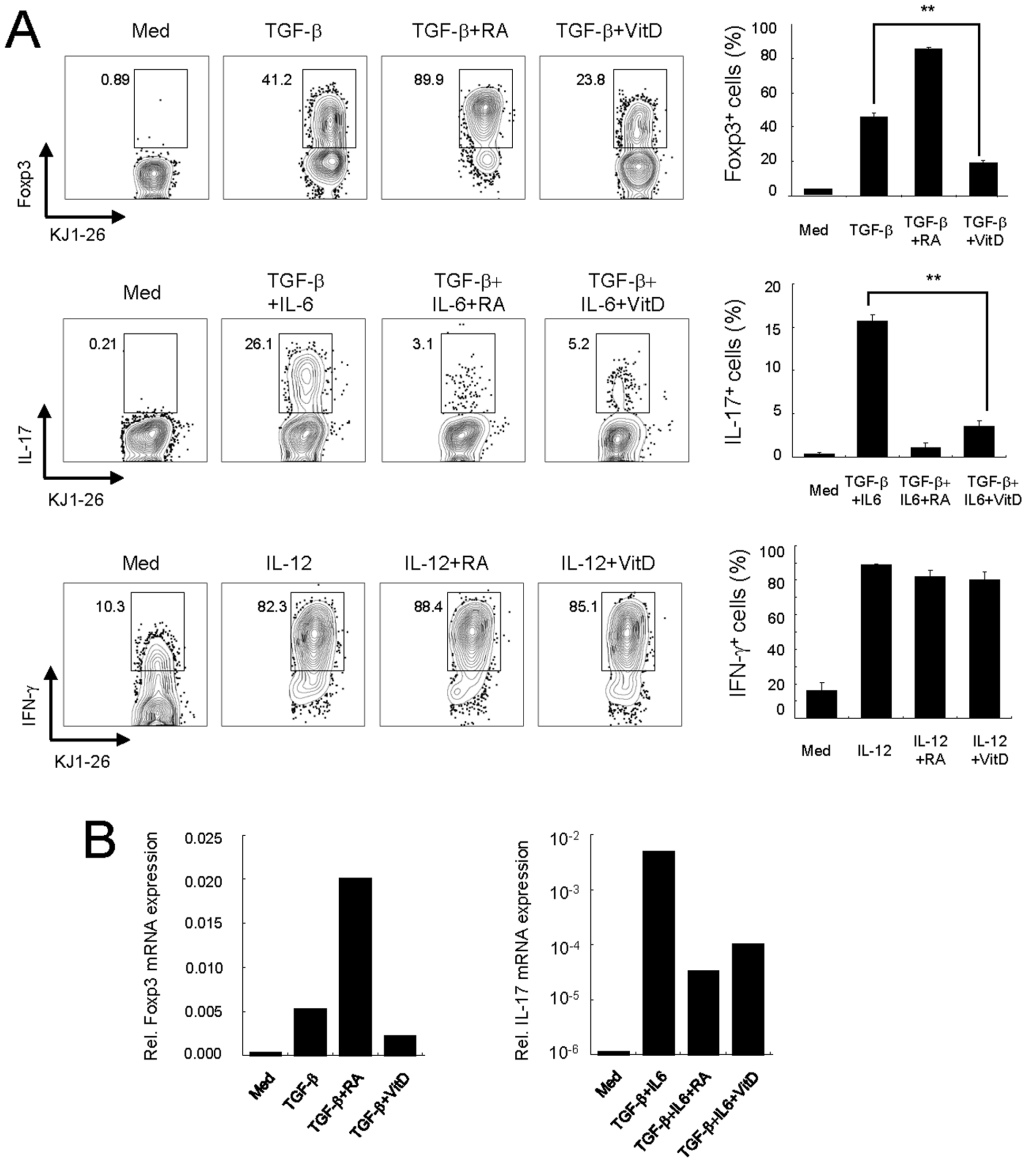
1,25(OH)_2_D_3_ negatively regulates Treg and T_H_17 induction in OVA-specific CD4^+^ T cells. (**A**) Naïve CD4^+^ T cells from Rag2^−/−^ DO11.10 mice (BALB/c background) were cultured with 0.25 µM OVA_323–339_ peptide in the presence of CD3^+^ T cell-depleted splenocytes for 4 days under polarizing conditions (Treg, T_H_17, or T_H_1) together with retinoic acid (RA, 100 nM) or 1,25(OH)_2_D_3_ (VitD, 100 nM) as described for Figure 2. Then cells were stained intracellularly for Foxp3, IL-17, or IFN-γ, respectively. The plots shown are gated on CD4^+^KJ1-26^+^ cells with quadrants drawn based on isotype controls. The numbers in the quadrants indicate cell percentages (left). Means ± SD of triplicate samples are plotted (right). Data are representative of five independent experiments with at least three mice per group. **p<0.01 compared with each cytokine-alone group. (**B**) Expression of Foxp3 and IL-17 genes was analyzed by quantitative PCR. Data are representative of five independent experiments with at least three mice per group.

We next examined the potential role of vitamin D_3_ on T_H_ generation by using well-established *in vitro* conditions. An *in vitro* treatment of 1,25(OH)_2_D_3_ on MOG-specific CD4^+^ T cells in the presence of MOG peptide, antigen-presenting cells (APCs), and TGF-β inhibited the expression of Foxp3 (Figure 2). Of note, 1,25(OH)_2_D_3_ also inhibited the generation of IL-17-secreting cells in the presence of TGF-β and IL-6 (Figure 2). In addition, since an inhibitory role of vitamin D_3_ on T_H_1 differentiation has been reported [35], we investigated the effect of 1,25(OH)_2_D_3_ under T_H_1 polarizing-conditions. However, the effect of 1,25(OH)_2_D_3_ on the differentiation of IFN-γ-secreting cells was not addressed in our system (Figure 2). To make clear whether 1,25(OH)_2_D_3_ can directly inhibit T_H_17 T cell differentiation regardless of antigen type, we used DO11.10 mice, which have OVA-specific CD4^+^ T cells. An *in vitro* culture of naïve KJ1-26^+^CD4^+^ T cells with 1,25(OH)_2_D_3_ in the presence of OVA peptide and APCs significantly inhibited the generation of both Foxp3 and IL-17-secreting cells (Figure 3A). Similar to MOG-specific CD4^+^ T cells, 1,25(OH)_2_D_3_ did not affect the differentiation of IFN-γ-secreting cells (Figure 3A). The mRNA levels of Foxp3 and IL-17 also declined in 1,25(OH)_2_D_3_-treated CD4^+^ T cells (Figure 3B). We also confirmed that 1,25(OH)_2_D_3_ inhibited Foxp3 and IL-17 expression in a dose-dependent manner (data not shown). Overall, our results demonstrate that vitamin D_3_ has a significant suppressive effect on Treg and T_H_17 generation but not on T_H_1 differentiation.

### Inhibition of Treg and T_H_17 differentiation by 1,25(OH)_2_D_3_ is dependent on the VDR on CD4^+^ T cells

**Figure 4:**
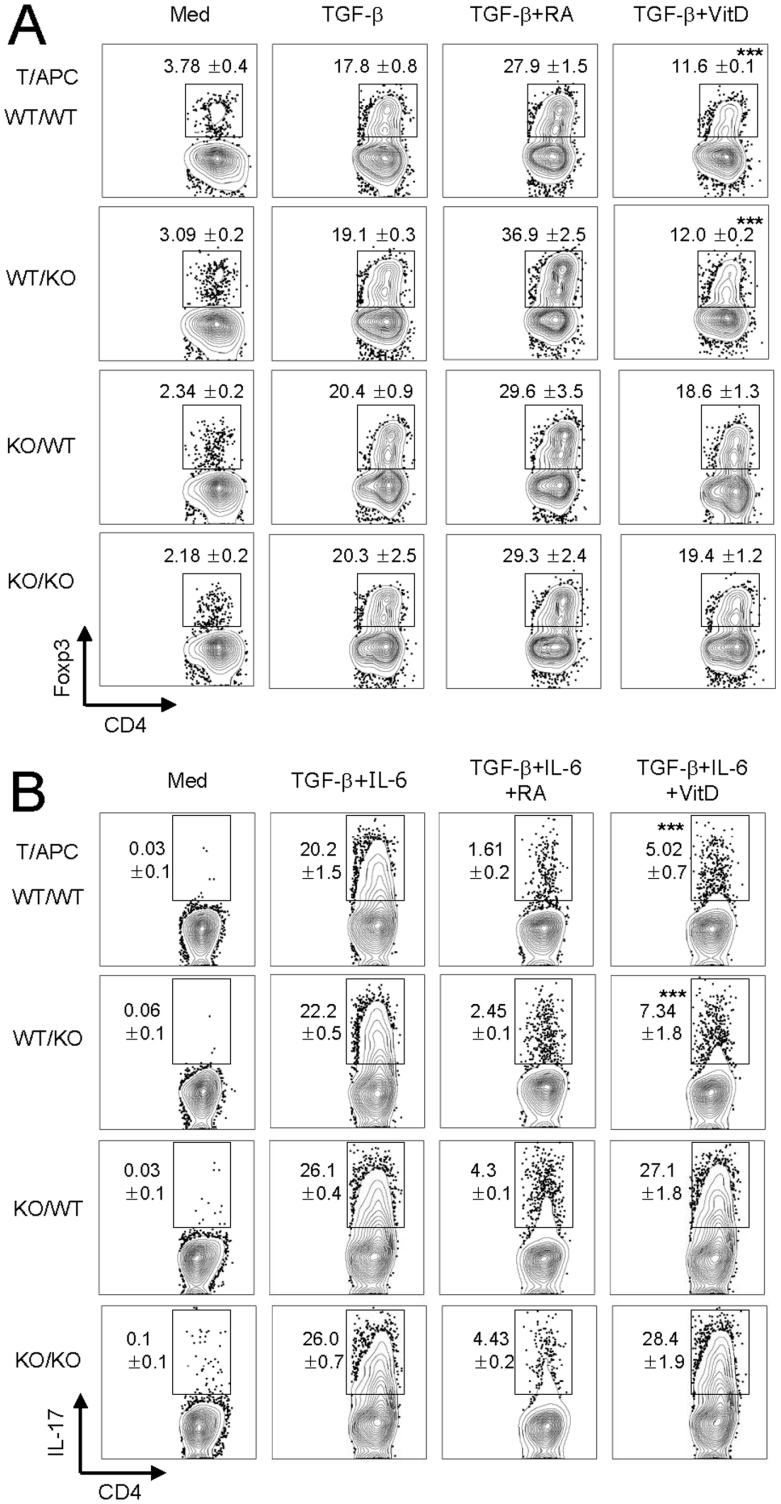
Vitamin D receptor on CD4^+^ T cells is required for regulation of Treg and T_H_17 differentiation by 1,25(OH)_2_D_3_. Purified naïve CD4^+^ T cells from wild-type (WT) or VDR^−/−^ (KO) mice of B6 background were cultured with APCs from WT or VDR^−/−^ mice in the presence of 1 µg/ml anti-CD3 mAb for 4 days under Treg-polarizing conditions (rTGF-β, 1 ng/ml; anti-IFN-γ, 10 µg/ml; and anti-IL-4, 10 µg/ml) or T_H_17-polarizing conditions (rTGF-β, 1 ng/ml; rIL-6, 20 ng/ml; anti-IFN-γ, 10 µg/ml; and anti-IL-4, 10 µg/ml). (**A**) Foxp3 expression in gated CD3^+^CD4^+^ cells was analyzed by flow cytometry. (**B**) For the IL-17A staining, CD4^+^ T cells were restimulated with PMA/ionomycin for 5 hr. Numbers beside quadrants indicate percentages of positive cells in each quadrant. Data are representative of three independent experiments with at least three mice per group. ***p<0.001 compared with cytokine-alone group.

The biological actions of vitamin D_3_ are mediated through the VDR, a member of the nuclear receptor superfamily [36]. To investigate whether VDR is essential for vitamin D_3_ to regulate T_H_ cell differentiation, we used VDR^−/−^ mice. As expected, deficiency of the VDR did not influence Treg and T_H_17 differentiation (Figure 4A and B). Of note, CD4^+^ T cells isolated from VDR^−/−^ mice were resistant to the inhibitory effect of vitamin D_3_ on the differentiation of Treg (Figure 4A) and T_H_17 (Figure 4B) under polarizing conditions. In contrast, the inhibitory role of 1,25(OH)_2_D_3_ on Treg and T_H_17 differentiation was still shown when VDR^−/−^ APCs were adopted (Figure 4A and B). Therefore, the VDR signal on activated CD4^+^ T cells was essential to down-regulate the development of Treg and T_H_17 cells.

### Down-regulation of Treg differentiation by 1,25(OH)_2_D_3_ is dependent on the low production of IL-2

**Figure 5:**
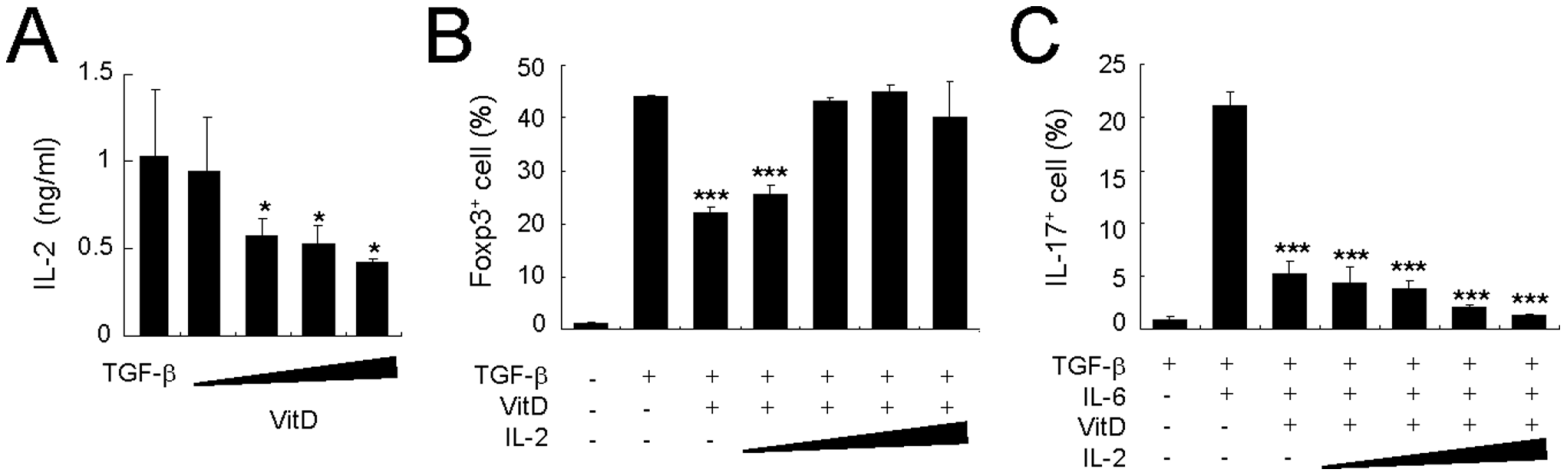
Exogenous IL-2 recovers the decreased Treg but not T_H_17 generation by 1,25(OH)_2_D_3_. Naïve CD4^+^ T cells from Rag2^−/−^ DO11.10 mice (BALB/c background) were cultured with 0.25 µM OVA_323–339_ peptide in the presence of CD3^+^ T cell-depleted splenocytes for 4 days. (**A**) Under Treg-polarizing conditions with 1,25(OH)_2_D_3_ (0.1, 1, 10, and 100 nM), culture supernatants were analyzed for IL-2 production by ELISA. (**B**) Under Treg-polarizing conditions, IL-2 cytokine was added in 1,25(OH)_2_D_3_-treated groups in a dose-dependent manner (IL-2: 0.1, 1, 10, and 20 ng/ml); 4 days later the CD4^+^ T cells were stained intracellularly for Foxp3. (**C**) Under T_H_17-polarizing conditions, 1,25(OH)_2_D_3_ (100 nM) and IL-2 (0.1, 1, 10, and 20 ng/ml) were added. The average frequency of IL-17A^+^ T cells in gated CD4^+^KJ1-26^+^ cells is shown. Means ± SD of triplicate samples are plotted. Data are representative of three independent experiments with at least three mice per group. *p<0.05, ***p<0.001 compared with cytokine-alone group.

Since vitamin D_3_ inhibits the secretion of IL-2, which is essential for the generation of Treg cells [37], [38], we first measured IL-2 levels in the culture supernatant after stimulation with vitamin D_3_. Interestingly, co-culture with vitamin D_3_ decreased IL-2 production by CD4^+^ T cells in a dose-dependent manner (Figure 5A). To investigate whether IL-2 recovers from the decrease of Treg differentiation caused by 1,25(OH)_2_D_3_, we added recombinant IL-2 (rIL-2) on the culture medium of CD4^+^ T cells in the presence of TGF-β and 1,25(OH)_2_D_3_. The addition of rIL-2 resulted in recovery of the Foxp3^+^ Treg cells that had been decreased by vitamin D_3_ compared with the numbers of Treg cells in the TGF-β-alone group (Figure 5B). However, in contrast to recovery of Treg cells following the addition of rIL-2, addition of rIL-2 did not reverse the inhibitory role of vitamin D_3_ on the generation of T_H_17 cells (Figure 5C). These results suggest that vitamin D_3_'s ability to decrease the number of Treg cells may be the result of its inhibitory effect on the amount of IL-2 secreted by CD4^+^ T cells.

### Regulation of T_H_17 differentiation by vitamin D_3_ is independent of IL-10

**Figure 6:**
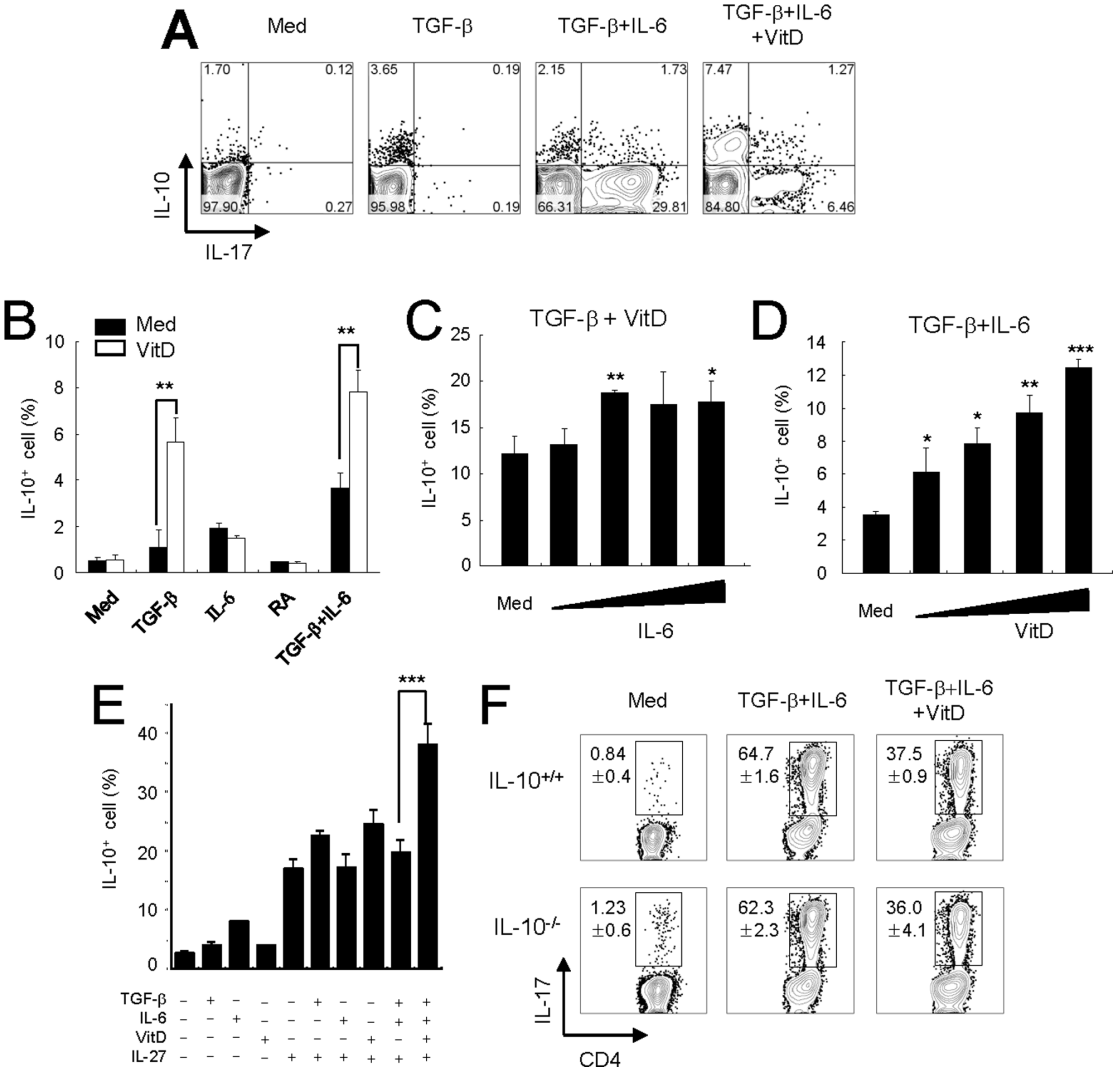
1,25(OH)_2_D_3_ and TGF-β plus IL-6 partially induce IL-10 production, but IL-10 is not involved in the inhibitory mechanism of vitamin D_3_. (**A** and **B**) Naïve CD4^+^ T cells from Rag2^−/−^ DO11.10 mice (BALB/c background) were cultured with OVA_323–339_ peptides in the presence of the indicated cytokines with and without 1,25(OH)_2_D_3_ (100 nM) for 4 days. The average frequency of IL-10-producing cells is shown. (**C**) The dose-dependent effect of IL-6 on IL-10 production in CD4^+^ T cells induced by TGF-β and 1,25(OH)_2_D_3_ was determined by titrated doses of IL-6 (0.1, 1, 10, and 100 ng/ml). (**D**) IL-10 production by CD4^+^ T cells cocultured with CD3-depleted splenocytes and OVA_323–339_ peptide was determined by titrated doses of 1,25(OH)_2_D_3_ (0.1, 1, 10, and 100 nM). (**E**) Average frequency of IL-10^+^cells among CD3^+^CD4^+^ cells as determined by flow cytometry after treatment with IL-27 and/or other indicated cytokines with or without 1,25(OH)_2_D_3_. Plots show mean ± SD of triplicate samples. *p<0.05, **p<0.01, ***p<0.001 compared with medium alone. (**F**) To analyze the effect of autocrine IL-10 on T_H_17 differentiation, we used IL-10^−/−^ mice of C57BL/6 background. Naïve CD4^+^ T cells isolated from IL-10^−/−^ or IL-10^+/+^ mice were stimulated with anti-CD3 mAb in the presence of the indicated condition for 4 days. IL-17 production in CD4^+^ T cells was analyzed by flow cytometry. Data are representative of three independent experiments with at least three mice per group.

Since a previous study showed that IL-10 plays a crucial role in the vitamin D_3_-mediated inhibition of EAE [32], we further assessed the role of IL-10 on the inhibition of IL-17 production by 1,25(OH)_2_D_3_ in activated T cells upon stimulation with TGF-β and IL-6. Treatment with 1,25(OH)_2_D_3_ alone did not increase the number of IL-10-producing T cells whereas co-treatment with TGF-β and 1,25(OH)_2_D_3_ led to a brisk increase in the number of IL-10-producing CD4^+^ T cells (Figure 6A and B), and co-treatment with IL-6 synergistically helped to produce IL-10 (Figure 6C). We then explored the dose-dependency of 1,25(OH)_2_D_3_ on IL-10 secretion under T_H_17-polarizing conditions. Treatment of CD4^+^ T cells with 1,25(OH)_2_D_3_ in the presence of TGF-β and IL-6 enhanced IL-10 production in a dose-dependent manner (Figure 6D).

Previous studies reported that IL-27 was up-regulated in APCs isolated from the CNS and lymph nodes of EAE-induced mice [39]. In addition, a combination of IL-27 and TGF-β has been shown to promote the differentiation of IL-10-producing Tr-1 cells [34], [40]. Therefore, it is possible that vitamin D_3_ might cooperate with IL-27 to suppress T_H_17 differentiation through IL-10. Interestingly, under T_H_17-polarizing conditions, treatment with a combination of IL-27 and 1,25(OH)_2_D_3_ generated a significantly higher number of IL-10-secreting cells when compared with the number of IL-10-secreting cells produced following treatment with IL-27 alone (Figure 6E). These data suggest that enhanced IL-10 production following treatment with vitamin D_3_ may regulate T_H_17 differentiation via an autocrine effect in the EAE inductive phase. To clarify the exact role of IL-10 in the suppression of T_H_17 differentiation by vitamin D_3_, we adopted IL-10^−/−^ mice. Under T_H_17-polarizing conditions, treatment with 1,25(OH)_2_D_3_ decreased IL-17 expression in T cells isolated from both IL-10^+/+^ and IL-10^−/−^ mice (Figure 6F). These results imply that IL-10 might not be directly involved in the suppressive role that vitamin D_3_ has on T_H_17 differentiation. Moreover, vitamin D_3_ may be a “helper” in the generation of IL-10-producing cells in an inflammatory environment but the effect of IL-10 is not essential for vitamin D_3_'s negative regulation of T_H_17 generation.

### The mechanism of suppression of T_H_17 generation by 1,25(OH)_2_D_3_ is independent on STAT1

**Figure 7:**
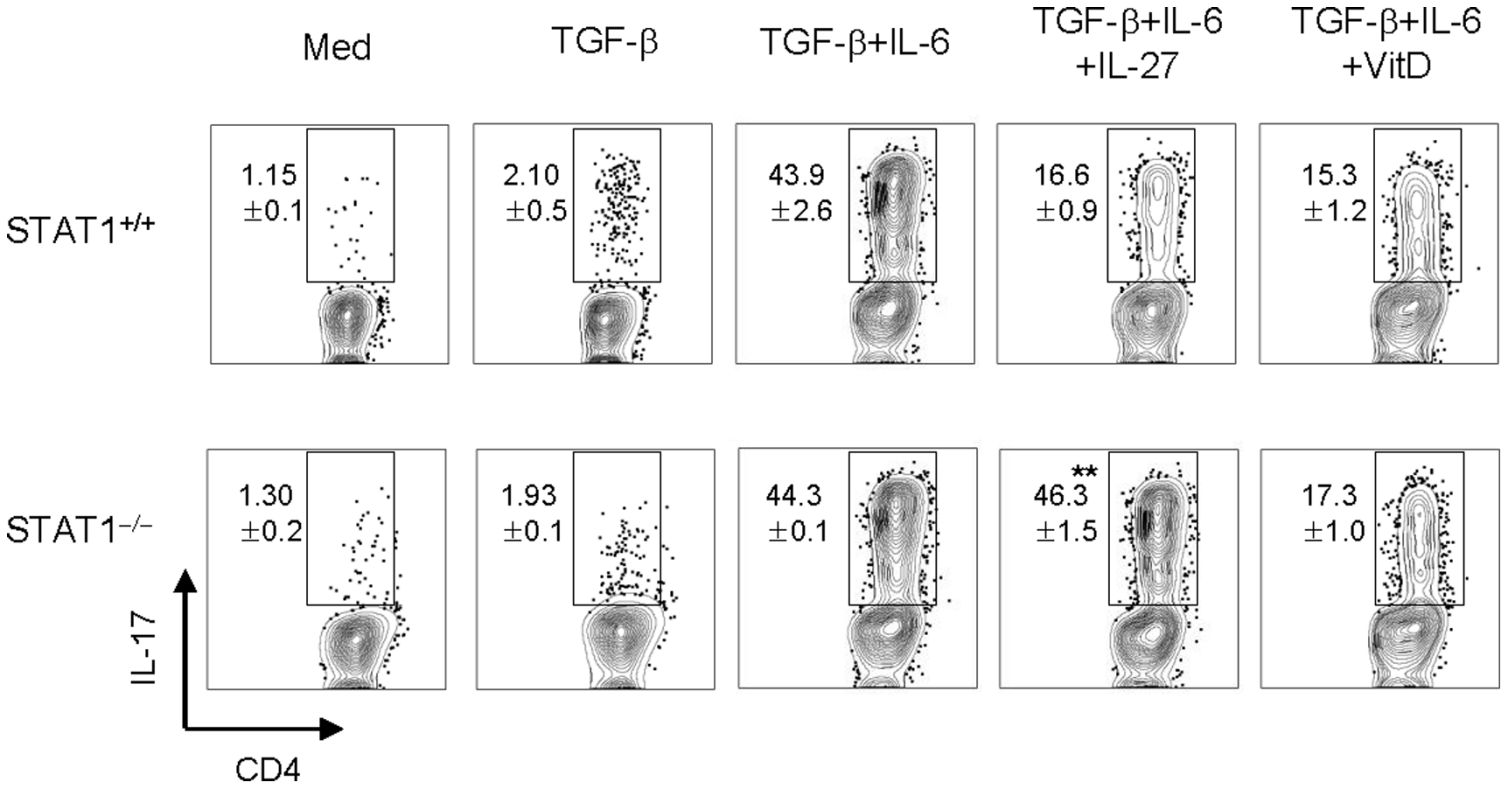
The inhibitory mechanism of 1,25(OH)_2_D_3_ is independent of the STAT1 signal. Naïve CD4^+^ T cells from STAT1^+/+^ and STAT1^−/−^ mice (B6 background) were cultured with anti-CD3 Abs (1 µg/ml) in the presence of CD3-depleted splenocytes for 4 days under various cytokine treatment conditions (IL-27, 10 ng/ml; TGF-β, 1 ng/ml; IL-6, 20 ng/ml; anti-IFN-γ, 10 mg/ml; or anti-IL-4, 10 mg/ml) with 1,25(OH)_2_D_3_ (100 nM) and then stained intracellularly for IL-17A and IL-10. Data are representative of three independent experiments with at least three mice per group. **p<0.01 compared with cytokine-alone group.

Since the effect of vitamin D_3_ is similar to that of IL-27, which inhibits the development of T_H_17 cells through STAT1-dependent mechanisms [41]–[43], we adopted STAT1^−/−^ mice to help us address the role that STAT1 signaling has on vitamin D3's inhibitory effect on T_H_17 differentiation. As expected, IL-27 failed to inhibit T_H_17 development in STAT1^−/−^ T cells under T_H_17-polarizing conditions (Figure 7). However, under T_H_17-polarizing conditions, 1,25(OH)_2_D_3_ suppressed IL-17 expression in both STAT1^−/−^ and STAT1^+/+^ CD4^+^ T cells (Figure 7). These results indicate that the negative regulation of T_H_17 by vitamin D_3_ is independent on STAT1.

### 1,25(OH)_2_D_3_ negatively regulates the expression and migration of CCR6^+^ T cells

**Figure 8:**
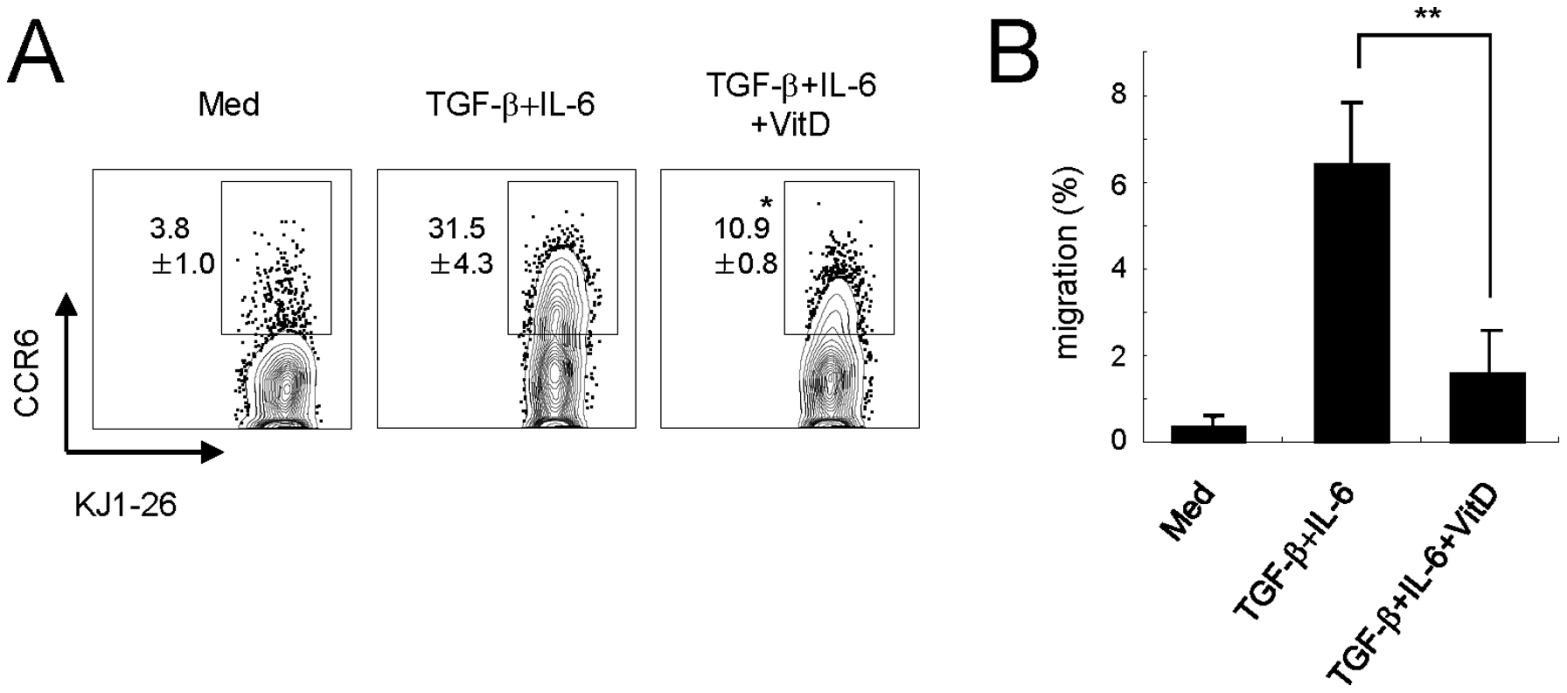
1,25(OH)_2_D_3_ inhibits the expression of the CCR6 molecule in activated T cells. (**A**) Flow cytometry analysis of CCR6 expression on activated T cells under T_H_17-polarizing conditions (as described for Figure 2). Data are representative of three independent experiments with at least three mice per group. *p<0.05 compared with cytokine-alone group. (**B**) MIP-3α/CCL20 was added to the lower chamber and *in vitro*-generated T_H_17 cells were applied to the upper chamber well. Two hours later, cells in the lower chamber were counted. Plots are mean ± SD of triplicate samples. Data are representative of two independent experiments with at least three mice per group. **p<0.01.

A recent study reported that the CCR6-CCL20 axis plays an essential role in controlling the entry of T_H_17 cells into the CNS and thus mediates the initiation of EAE [44]. In our present study, we found significantly reduced migration of CD4^+^ T cells into the CNS following oral feeding of 1,25(OH)_2_D_3_ (Figure 1B). To investigate the direct effect of vitamin D_3_ on the migration of T_H_17 cells into the CNS, we analyzed the CCR6 expression of the OVA-specific CD4^+^ T cells under T_H_17-polarizing conditions. Interestingly, 1,25(OH)_2_D_3_ directly inhibited CCR6 expression in the presence of TGF-β and IL-6 (Figure 8A). We further checked the expression levels of CCR6 in an EAE-relevant T cell system. Interestingly, 1,25(OH)_2_D_3_ reduced the expression of CCR6 on activated MOG-specific CD4^+^ T cells (data not shown). To further address the regulation of CCR6 expression by 1,25(OH)_2_D_3_, we evaluated the migratory characteristics of T_H_17 cells generated *in vitro* using the Transwell chemotaxis assay. Interestingly, we found that T_H_17 cells elicited by TGF-β and IL-6 signals migrated principally toward MIP-3α/CCL20 (Figure 8B). Of note, consistent with the suppression of CCR6 expression by 1,25(OH)_2_D_3_, 1,25(OH)_2_D_3_-treated T_H_17 cells migrated, to a much lesser degree, toward MIP-3α/CCL20 (Figure 8B). These results suggest that vitamin D_3_ inhibits the CCR6 expression on T_H_17 cells, which may block T_H_17 cells from entering the CNS.

## Discussion

In this study, we found that oral administration with 1,25(OH)_2_D_3_ significantly reduced the number of lymphocytes in the CNS of EAE-induced mice. The active form of vitamin D_3_ is a direct inhibitor for T_H_17 differentiation via the VDR signal but works independently of IL-2, IL-10, and STAT1 signals *in vitro*. In addition, we studied whether vitamin D_3_ negatively regulate the expression of IL-6R to inhibit T_H_17 differentiation but we did not see any significant differences in the IL-6R expression of CD4^+^ T cells after co-culture with IL-6, TGF-β and vitamin D_3_ (data not shown). Most importantly, 1,25(OH)_2_D_3_ negatively regulates the expression of CCR6 on the T_H_17 cells. Recently the CCR6-CCL20 axis was reported to play an essential role in controlling the entry of T_H_17 cells into the CNS, thus mediating the initiation of EAE [44]. Our data suggest the possibility that VDR activation modulates CCR6 expression and leads to a functional hypo-responsiveness to CCL20. Overall, our current results imply that oral administration of vitamin D_3_ could be an effective tool for the treatment of T_H_17-mediated autoimmune diseases.

Several recent studies reported the immunomodulatory effects of vitamin D_3_ on the differentiation and function of Treg cells, specifically the ability of topically applied vitamin D_3_ to increase the suppressive activity of Treg cells and the *in vivo* expansion of antigen-specific Treg cells following the topical application of calcipotriol, as a vitamin D_3_ analog [45], [46]. In addition, vitamin D_3_-treated DCs induce Treg cells via independence of an inhibitory receptor immunoglobulin-like transcript 3 (ILT3) molecule, which is required for induction of Treg [47]. These studies suggest that topical application of vitamin D_3_ might alter DC function in the periphery and affect the differentiation and functions of Treg cells. In contrast, our present data show that the expression of TGF-β mediated Foxp3 was inhibited by 1,25(OH)_2_D_3_ via the VDR signal on CD4^+^ T cells (Figure 4). In particular, *in vitro* treatment of 1,25(OH)_2_D_3_ resulted in decreased levels of IL-2 production by activated CD4^+^ T cells in concurrence with prior reports [48]–[50]. Thus, IL-2 might be crucial for inhibiting Treg differentiation by vitamin D_3_.

Although IL-2 blocks the inhibitory role of 1,25(OH)_2_D_3_ on Treg generation, 1,25(OH)_2_D_3_ and IL-2 synergistically constrain IL-17 production in CD4^+^ T cells (Figure 5). Thus, it seems likely that the mechanisms by which 1,25(OH)_2_D_3_ inhibits the generation of Treg and T_H_17 cells differ. The inhibitory effect of vitamin D_3_ seems to be similar to that of IL-27, which inhibits the lineage commitment of T_H_17 cells [33, 41–43 51] and induces IL-10 production, which, in turn, suppresses EAE initiation [34]. Since the ability of IL-27 to block the generation of T_H_17 cells is dependent on the transcription factor STAT1 [41]–[43], we next sought to determine whether STAT1 is involved in 1,25(OH)_2_D_3_-mediated inhibitory effects on the development of T_H_17 cells. However, unlike IL-27, 1,25(OH)_2_D_3_'s ability to inhibit the development T_H_17 cells was independent on the STAT1.

A previous study demonstrated that Smad3, signal transducers of the TGF-β superfamily, mediated cross-talk between TGF-β and vitamin D_3_ signaling pathways [52]. The cooperative actions of the Smad3-VDR complex can be synergistic or antagonistic in a conditional manner [53]. In addition, another study suggested that the enhancement of TGF-β-driven Smad3 signaling by retinoic acid increases the number of Foxp3-expressing T cells and inhibits the development of T_H_17 cells [54]. These several lines of study lead us to speculate that Smad3 mediates vitamin D_3_'s ability to inhibit the development of T_H_17 cells. However, as of yet we have not been able to verify this hypothesis.

Another study reported that the combination of vitamin D_3_ and dexamethasone increased the frequency at which IL-10-producing regulatory T cells are generated [55]. Further, vitamin D_3_ failed to inhibit EAE in IL-10^−/−^ or IL-10R^−/−^ B6 mice [32]. However, in our *in vitro* study, vitamin D_3_ alone failed to induce IL-10 production in activated T cells (Figure 6A and B). Thus, it requires additional factors to protect against EAE through the IL-10 effect. Vitamin D_3_ helped TGF-β mediate IL-10 production and strongly enhanced the generation of IL-27-mediated IL-10-producing CD4^+^ T cells in an *in vitro* system (Figure 6E). A recent study clearly showed that IL-27 plays a crucial role in the development of IL-10-producing anti-inflammatory T cells [40]. Others reported that IL-27 and IL-27R are up-regulated in APCs from the CNS and lymph nodes in EAE-induced mice [39]. When considered together, the facts that IL-27 is a good inducer of IL-10-producing T cells and that 1,25(OH)_2_D_3_ possesses synergistic effects under T_H_17-polarizing conditions suggest that vitamin D_3_ requires the presence of TGF-β and IL-6 to increase the number of IL-27-mediated IL-10-producing T cells. Thus, it is possible that vitamin D_3_ cooperates with IL-27 to protect against EAE through IL-10.

A recent study found that CCR6 plays an essential role in the initiation of EAE and that CCL20, a CCR6 ligand, is constitutively expressed in choroid plexus epithelial cells in mice and humans [44]. Further, T_H_17 cells predominantly express CCR6 [56]. In accordance, it has been suggested that the recruitment of T_H_17 cells via the CCR6-CCL20 axis is necessary for development of T_H_17 cell-mediated autoimmune disease. As depicted in Figure 1B, CD4^+^ T cells were highly infiltrated in EAE-induced mice whereas 1,25(OH)_2_D_3_-treated mice had extremely low numbers of CD4^+^ T cells in their CNS. However, although CCR6 are important for recruitment of T_H_17 cells into the mouse CNS, this has not yet been shown in human MS. Rather IL-17 and IL-22 receptors on blood-brain barrier endothelial cells play a crucial role on ICAM-1-mediated migration of T_H_17 in MS [8], [11]. Further study is required to elucidate differences between mouse and human receptors.

We raised two hypotheses to explain the absence of lymphocytes in the CNS after vitamin D_3_ treatment. First, we postulated that vitamin D_3_ causes lymphocyte death; however, vitamin D_3_ did not induce apoptosis and/or cell death of activated T cells under T_H_17-polarizing conditions (data not shown). Our second hypothesis was that regulation of T_H_17 cell recruitment occurs via chemokine and chemokine receptors. As expected, we found that 1,25(OH)_2_D_3_ inhibited the expression of CCR6 on T cells that had been activated by both TGF-β and IL-6 (Figure 8). Since one recent study also showed that vitamin D_3_ induces the expression of CCR10 on activated CD4^+^ T cells in the presence of IL-12 [57], we investigated the possibility that vitamin D_3_ also plays a role in the ability of T_H_17 cells to express CCR10 instead of CCR6. Those investigations showed that 1,25(OH)_2_D_3_ did not induce CCR10 expression on the T_H_17 cells in the presence of TGF-β and IL-6 (data not shown). Overall, we found that vitamin D_3_ down-regulates CCR6 but not CCR10 expression in the T_H_17-conditioned circumstance.

In summary, our study results suggest that vitamin D_3_ can directly regulate T cell development and migratory function. The VDR signal on the CD4^+^ T cells inhibits the expression of IL-17, IL-2, Foxp3, and CCR6 but enhances the expression of IL-10. These characteristic features of vitamin D_3_ could be applied to preventive and therapeutic strategies for T_H_17-mediated autoimmune diseases.

## Materials and Methods

### Mice

Female BALB/c and C57BL/6 mice (Charles River Laboratories, Seoul, Korea) were used at ages 8–12 wks. Rag2^−/−^ DO11.10 mice (BALB/c background), MOG-TCR (2D2) transgenic mice (B6 background), IL-10^−/−^ mice (B6 background), and STAT1^−/−^ (B6 background) were purchased from Taconic (Germantown, NY) and Jackson Laboratory (Bar Harbor, ME). VDR^−/−^ mice were kindly provided by Prof. S. Kato (University of Tokyo, Tokyo, Japan). All mice were maintained under pathogen-free conditions in the experimental facility at the International Vaccine Institute (Seoul, Korea) where they received sterilized food and water *ad libitum* and all experiments described in this article were approved by Institutional Animal Care and Use Committees (Approval No: PN 0901).

### Vitamin D_3_ treatment and induction of EAE

One mg/ml stock of 1,25(OH)_2_D_3_ (Sigma-Aldrich, St. Louis, MO) in DMSO was added to water (50 ng/day for females; 100 ng/day for males). Alternatively, 200 ng of 1,25(OH)_2_D_3_ in oil or oil only as a placebo was injected i.p. [32]. To induce EAE, myelin oligodendrocyte glycoprotein peptide (MOG_33–55_, MEVGWYRSPFSRVVHLY-RNGK) was resuspended in sterile PBS to a concentration of 4 mg/ml and then emulsified with an equivalent volume of complete Freund's adjuvant (CFA) supplemented with 5 mg/ml *Myocobacterium tuberculosis* H37Ra (BD Diagnostic Systems, Sparks, MD). EAE was induced in 9- to 10-wk old female C57BL/6 mice by s.c. injection of 100 µl of MOG_35–55_/CFA homogenate delivering 200 µg of MOG_35–55_ peptide. On days 1 and 3 after immunization, the mice were injected i.p. with 200 ng of pertussis toxin (Sigma-Aldrich) diluted in PBS. The mice were then scored daily for clinical signs of EAE using the following scale for a “disease score”: 0 =  no clinical disease, 1 =  loss of tail tone, 2 =  unsteady gait, 3 =  hind limb paralysis, 4 =  forelimb paralysis, 5 =  death.

### *In vitro* T_H_ generation

All experiments were performed with highly purified CD4^+^CD25^−^ naïve T cells (>95% purity). To purify naïve T cells, erythrocyte-depleted splenocytes were first depleted of CD25^+^ cells via magnetic selection using anti-CD25 microbeads (Miltenyi Biotec, Auburn, CA). In the remaining population, CD4^+^ cells were positively selected using anti-CD4 microbeads (Miltenyi Biotec). Cells were cultured in complete RPMI 1640 supplemented with 10% FBS and 50 U/ml of penicillin and streptomycin. For antigen-specific stimulation, purified CD4^+^ T cells from MOG TCR-Tg or Rag2^−/−^ DO11.10 mice were incubated with MOG_35–55_ (25 µg/ml) or OVA_323–339_ (0.2 µM) peptide presented by CD3-depleted splenocytes under Treg-polarizing conditions (1 ng/ml rhTGF-β1, 10 µg/ml anti-IFN-γ, and 10 µg/ml anti-IL-4); under T_H_17-polarizing conditions (1 ng/ml rhTGF-β1, 20 ng/ml rmIL-6, 10 µg/ml anti-IFN-γ, and 10 µg/ml anti-IL-4); or under T_H_1-polarizing conditions (4 ng/ml rmIL-12 and 10 µg/ml anti-IL-4). Death cells were confirmed by propidium iodide (PI; BD Pharmingen, San Diego, CA) staining and were excluded before analysis.

### Flow-cytometric analyses

CD4^+^ T cells were collected and stimulated with PMA (50 ng/ml; Sigma-Aldrich) and ionomycin (750 ng/ml; Calbiochem, La Jolla, CA) for 5 hr in the presence of Golgi Plug (BD Pharmingen). Anti-mouse CD3e-PerCP (145-2C11; BioLegend, San Diego, CA), anti-mouse CD4-FITC (RM4-5; BD Pharmingen), anti-mouse DO-11.10 Clonotypic TCR (KJ1-26; BD Pharmingen), anti-mouse TCR Vα3.2-FITC (RR3-16; BD Pharmingen), anti-mouse TCR Vβ 11 PE (RR3-15; BD Pharmingen), anti-mouse IL-17A-APC (eBio17B7; eBioscience, San Diego, CA), anti-mouse IFN-γ-APC (XMG1.2; BD Pharmingen), anti-mouse Foxp3-APC (FJK-16s; eBioscience), and anti-mouse IL-10-PE Abs (JES5-16E3; BD Pharmingen) were used according to manufacturers' instructions. Data were obtained using a FACSCalibur (BD Immunocytometry Systems, San Jose, CA) with CellQuest software and the profiles were analyzed using Flowjo flow cytometry software (TreeStar Inc., Ashland, OR).

### Real-time PCR and RT-PCR

To assess the expression of IL-17 and Foxp3, mRNA was extracted using TRIzol (Invitrogen, Camarillo, CA) according to the manufacturer's instructions and then reverse transcribed into cDNA. The primer sequences for amplification of each transcript are as follows: IL-17, 5′-GGTCAACCTCAAAGTCTTTAACTC-3′ and 5′-TTAAAAAT GCAAGTAA GTTTGCTG-3′; Foxp3, 5′-CAGCTGCCTACAGTGCCCCTAG-3′ and 5′-CATTTGC CAGCAGTGGGTAG-3′; β-actin, 5′- ATCTGGCACCACACCTTCTACAATGAGCT GCG-3′ and 5′-CGTCATACTCCTGCTTGCTGATCCACAT CTGC-3′.

### Chemotaxis assay

To evaluate the migration of T_H_17 cells, 5-µm Transwell inserts (Corning, Cambridge, MA) containing 1×10^5^ *in vitro*-generated T_H_17 cells were placed in the 24-well plate so as to make contact with 600 µl of the medium alone (basal) or with 100 nM MIP-3α/CCL20 (R&D Systems, Minneapolis, MN). Two hours later, the inserts were removed and the population that migrated to the well bottoms was counted.

### Statistics

Data are expressed as the mean ± SD. Statistical comparisons between experimental groups were performed using the Student *t*-test.
