# Correction: 1,25-Dihydroxyvitamin D_3_ Inhibits the Differentiation and Migration of T_H_17 Cells to Protect against Experimental Autoimmune Encephalomyelitis

**DOI:** 10.1371/annotation/6da7b65e-dda2-467d-bcb3-82d5669f6bc6

**Published:** 2010-12-02

**Authors:** Jae-Hoon Chang, Hye-Ran Cha, Dong-Sup Lee, Kyoung Yul Seo, Mi-Na Kweon

The following information was left out of the funding section: "This work was supported by a grant of the Korea Healthcare technology R&D Project, Ministry for Health, Welfare & Family Affairs, Republic of Korea (A084790).”

